# Post-translational governance of NF-κB in cancer immunity: mechanisms and therapeutic horizons

**DOI:** 10.3389/fimmu.2025.1627084

**Published:** 2025-09-16

**Authors:** Yue Hong, Ying Fu, Qian Long

**Affiliations:** ^1^ Department of General Surgery, The Second Xiangya Hospital, Central South University, Changsha, China; ^2^ Clinical Research Centre For Breast Disease in Hunan Province, Changsha, China; ^3^ Department of Nephrology, Hunan Key Laboratory of Kidney Disease and Blood Purification, Institute of Nephrology, The Second Xiangya Hospital at Central South University, Changsha, China; ^4^ Postdoctoral Mobile Station of Basic Medical Sciences, The Second Xiangya Hospital of Central South University, Changsha, Hunan, China

**Keywords:** NF-κB signaling, post-translational modifications, Tumor immune microenvironment, immune evasion, immunotherapy resistance, precision immunotherapy

## Abstract

Nuclear factor-κB (NF-κB) is a central transcriptional orchestrator of inflammation, immune modulation, and tumor progression. Beyond canonical signal transduction, the immunological functions of NF-κB are intricately governed by a spectrum of post-translational modifications (PTMs)—including phosphorylation, acetylation, ubiquitination, and methylation—that fine-tune its activation, nuclear translocation, DNA binding, and transcriptional specificity. In this Review, we explore how these context-dependent PTMs dynamically shape NF-κB’s role in cancer immunity: promoting macrophage polarization, controlling antigen presentation by dendritic cells, regulating T cell exhaustion, and sustaining immunosuppressive networks within the tumor microenvironment. We further delineate how PTM-mediated NF-κB signaling interfaces with immune checkpoint expression—particularly PD-L1 and IDO1—and fuels resistance to immunotherapies. Emerging pharmacological strategies targeting NF-κB-modifying enzymes or degradation via PROTACs hold promise to reprogram the immune landscape. By integrating mechanistic insight with translational potential, we position NF-κB’s post-translational regulation as a fertile axis for next-generation immunotherapeutic innovation.

## 1 Introduction

Nuclear factor-kappa B (NF-κB) signaling lies at the epicenter of immune regulation in cancer, functioning as a molecular switchboard that integrates external inflammatory cues with internal transcriptional responses (1, 2). Over the past decades, extensive studies have delineated its canonical and non-canonical pathways (3, 4), yet the nuanced modulation of NF-κB activity via post-translational modifications (PTMs) remains an evolving frontier with profound implications for tumor immunology (5, 6). PTMs serve not only to activate or silence NF-κB but also to reprogram its target gene repertoire, dictating cell-specific immune responses (7).

In the context of malignancy, dysregulated PTMs of NF-κB subunits orchestrate a range of immune remodeling events—from fostering chronic inflammation and immunosuppressive phenotypes to dampening antigen processing and facilitating immune escape (8). Given the centrality of these processes to tumor progression and immunotherapeutic response, dissecting the PTM landscape of NF-κB offers a powerful lens through which novel intervention points can be identified. Despite decades of research, critical gaps remain in our understanding of NF-κB’s pan-cancer dynamics. Key questions include how NF-κB activity is differentially regulated across immunological landscapes, and how pathway targeting can be fine-tuned for maximal clinical benefit (9).

This Review synthesizes current advances in our understanding of NF-κB PTMs and their consequences on cancer immunity, highlighting emerging therapeutic strategies that leverage this axis to restore anti-tumor immune competence. We propose that decoding the PTM architecture of NF-κB will be pivotal for refining immune checkpoint blockade, overcoming resistance, and designing next-generation immunomodulators.

## 2 PTMs in the NF-κB signaling pathway

### 2.1 Phosphorylation

RelA can be phosphorylated both in the cytoplasm and nucleus. In the head and neck squamous cell carcinoma, phosphorylation at S276 enhances NF-κB transcriptional activity and promotes NF-κB–dependent expression of cytokines such as IL-6 and IL-8 (10). Phosphorylation at S536 activates the canonical NF-κB pathway and mediates malignant proliferation of cancer cells. Phosphorylation at S529 has also been described to moderately enhance NF-κB transcriptional activity. Studies have shown that, in pancreatic ductal adenocarcinoma, phosphorylation mediated by Polo-like kinase 1 (Plk1) can inhibit the nuclear translocation of NF-κB, thereby reducing PD-L1 expression (11).

### 2.2 Acetylation

Acetylation is another important post-translational modification of RelA, primarily occurring in the nucleus, and it plays multiple regulatory roles in NF-κB activity. For example, acetylation at lysine K221 enhances the DNA-binding ability of NF-κB (12). Acetylation at K310 is essential for the full transcriptional activity of NF-κB. A study reported that, in pancreatic ductal adenocarcinoma, histone deacetylase 5 (HDAC5) suppresses the transcriptional activity of NF-κB by directly interacting with p65 and mediating deacetylation at K310, thereby regulating PD-L1 expression (13).

### 2.3 Ubiquitination

Ubiquitination of NF-κB is also an important mechanism for regulating its activity. SOCS1, an E3 ligase for the RelA subunit, binds to RelA and mediates its ubiquitination, thereby inhibiting NF-κB activity. Ubiquitination mediated by PDLIM2 can also impact RelA function (14). In hepatocellular carcinoma, inhibition of NF-κB ubiquitination has been shown to promote tumor immune evasion (15).

### 2.4 SUMOylation

SUMOylation is a post-translational modification involving the covalent attachment of small ubiquitin-like modifiers (SUMOs) to lysine residues on target proteins. Studies have shown that, in macrophages, TRIM60-mediated SUMOylation of TAB2 at K329 and/or K562 suppresses downstream NF-κB activation, leading to reduced production of pro-inflammatory cytokines and attenuated TLR-mediated innate immune responses *in vivo* (16) (**Table 1**).

**Table 1:** Overview of NF-κB-associated PTMs and their roles in tumor immunity.

Modification	Tumours	Effect	Reference
Phosphorylation	PDAC	inhibition of the translocation of NF-κB	(11)
Acetylation	PDAC	suppression the transcriptional activity of NF-κB	(13)
Ubiquitination	HCC	tumor immune evasion	(15)
SUMOylation	/	attenuated innate immune responses	(16)

## 3 PTM-governed NF-κB signaling in the tumor microenvironment

The tumor microenvironment (TME) is a dynamic and immunologically active niche that not only responds to but actively shapes tumor evolution, immune escape, and resistance to therapy. Comprised of neoplastic cells, infiltrating immune subsets, stromal fibroblasts, vascular elements, and a plethora of soluble factors, the TME is continually reprogrammed through signaling cascades—chief among them, NF-κB. While extensively studied for its canonical role in sustaining tumor cell survival and proliferation, NF-κB also serves as a central modulator of immune dynamics within the TME (17).

Recent studies highlight that the immunoregulatory potential of NF-κB is not solely dictated by pathway activation, but rather by the combinatorial effects of post-translational modifications (PTMs) that modulate its stability, transcriptional specificity, and co-factor recruitment. Phosphorylation of p65 at Ser276 enhances its interaction with CBP/p300, promoting pro-inflammatory gene transcription; conversely, acetylation at Lys122 or Lys123 represses DNA binding and nuclear retention, limiting NF-κB activity (18, 19). These PTMs function as molecular rheostats, enabling NF-κB to fine-tune the expression of cytokines, chemokines, and immune checkpoint molecules in a context-specific manner.

NF-κB-driven transcription of TNF-α and IL-6 exemplifies its dualistic immunological functions. TNF-α may promote cytotoxic T cell recruitment or paradoxically support tumor survival via NF-κB-dependent feedback loops, depending on the cellular context and PTM patterning of NF-κB subunits (20, 21). IL-6, a major STAT3 activator, facilitates immune evasion and suppresses dendritic cell maturation, reinforcing a tolerogenic TME (22, 23). Chemokines under NF-κB control, including CCL2 and CXCL8, orchestrate the recruitment of myeloid-derived suppressor cells (MDSCs) and tumor-associated macrophages (TAMs), sustaining chronic inflammation and amplifying immunosuppressive circuits (24–26). Importantly, NF-κB activation—particularly when potentiated by SUMOylation or specific lysine acetylation events—directly upregulates PD-L1 expression on both tumor and immune cells, diminishing T cell cytotoxicity and contributing to immune checkpoint blockade resistance (27). H3K18la lactylation directly promotes NF-κB signaling by enhancing the binding affinity to the promoter regions of Rela and NFκB1, leading to increased expression of IL-6 and IL-8 (28). These findings suggest that the PTM landscape of NF-κB may dictate whether it fosters immune activation or suppression.

At the cellular level, NF-κB’s impact is cell-type specific and PTM-dependent. In TAMs, NF-κB signaling—modulated by methylation of RelA—can skew polarization toward an M2-like phenotype that supports angiogenesis and immune tolerance, although certain phosphorylation events promote M1-like, pro-inflammatory programs under stress conditions (29, 30). In T cells, NF-κB activity governs activation thresholds, effector differentiation, and exhaustion via crosstalk with PD-1 and TIM-3 signaling pathways (31).

Thus, NF-κB functions as a molecular fulcrum balancing anti-tumor immunity and immune evasion. Its functional output is not binary, but dictated by a dynamic PTM code that integrates environmental cues within the TME. Dissecting this PTM-governed signaling landscape offers a refined framework for designing precision immunotherapies that aim to selectively reprogram the immune milieu toward sustained tumor control.

## 4 PTM-regulated NF-κB signaling in immune cell subsets across cancers

### 4.1 Macrophage polarization

Tumor-associated macrophages (TAMs) display remarkable plasticity, shifting between pro-inflammatory M1 and anti-inflammatory M2 phenotypes depending on environmental and molecular cues. NF-κB is a central determinant of this polarization, yet its activity is governed by distinct post-translational modifications (PTMs) that dictate transcriptional outcomes. Upon stimulation by LPS or IFN-γ, NF-κB subunits undergo phosphorylation (e.g., p65 Ser536) and acetylation that favor M1-associated gene expression, promoting the secretion of TNF-α, IL-1β, and IL-6 and sustaining antitumor inflammation (32, 33). Conversely, IL-4–induced acetylation or SUMOylation patterns on NF-κB facilitate IL-10 and TGF-β expression, favoring M2 polarization and immune suppression (34). In the immunosuppressive TME, PTM-skewed NF-κB signaling often biases macrophages toward the M2 phenotype (35).

NF-κB-driven polarization varies across cancer types. In oral squamous cell carcinoma, NF-κB activation enhances M1 macrophage responses (36), whereas in lung and colorectal cancers, PTM-modulated NF-κB drives M2 TAM enrichment and links chronic inflammation to tumor progression (37–39). Broadly, elevated NF-κB activity—particularly in the context of activating PTMs—correlates with M2 TAM predominance in breast, ovarian, prostate, pancreatic, and gastric cancers (40–45).

### 4.2 Dendritic cells and antigen presentation

Dendritic cells (DCs) are pivotal in orchestrating antitumor T cell responses. Canonical NF-κB activation via MyD88 and TLRs promotes DC maturation by enhancing expression of CD80, CD86, and cytokines like IL-12. Specific PTMs—such as p65 phosphorylation or methylation—amplify DC immunostimulatory capacity by upregulating MHC-I and MHC-II presentation pathways (46–49). cDC1s cross-present tumor antigens to CD8^+^ T cells, whereas cDC2s prime CD4^+^ T cell subsets. NF-κB PTMs fine-tune these processes, dictating the balance between effective priming and tolerance.

Functionally, cDC1s excel at cross-presenting tumor antigens on MHC-I to CD8^+^ T cells, initiating robust cytotoxic responses (50), while cDC2s promote CD4^+^ T cell polarization through MHC-II pathways, modulating Th1, Th2, and Th17 responses. NF-κB activation enhances DC function by amplifying MHC molecule expression and sustaining the ability to prime T cells. However, chronic NF-κB activation—often seen in inflammation-driven tumors like colorectal or gastric cancer—may paradoxically impair DC functionality, favoring tumor progression despite increased APC recruitment (51–53).

### 4.3 T cell subset regulation

For CD8^+^ T Cells, NF-κB signaling is essential for CD8^+^ T cell activation, expansion, and effector function. TCR engagement and CD28 co-stimulation trigger NF-κB activation, promoting IL-2, IFN-γ, and granzyme B expression. PTMs such as RelA acetylation or p50 ubiquitination determine whether NF-κB drives cytotoxic responses or upregulates inhibitory checkpoints like PD-1 (54–57). In the TME, this dual role of PTM-modulated NF-κB contributes to CD8^+^ T cell exhaustion. For Regulatory T Cells (Tregs), Tregs rely on NF-κB for lineage stability and suppressive function, with Foxp3 expression directly regulated by c-Rel and RelA. Loss of PTM-regulated NF-κB activity (e.g., c-Rel phosphorylation) impairs Treg development, while sustained NF-κB activation enhances Treg infiltration and immunosuppression within tumors (58–61). Studies have shown that histone lactylation may play a regulatory role in the expression of NF-κB target genes (62). In TME, Treg cells can absorb lactate and regulate NF-κB p65–mediated gene transcription through histone H3K18 lactylation (H3K18la). This process upregulates the expression of TNFR2 and immunosuppressive molecules on Treg cells, thereby enhancing their immunosuppressive function against CD8^+^ T cells (63). For CD4^+^ T Helper Cells, NF-κB regulates CD4^+^ T cell fate via PTM-controlled activation of transcription factors. RelA and RelB induce T-bet and IFN-γ to promote Th1 polarization, while c-Rel and acetylation-dependent signaling favor GATA3-driven Th2 differentiation (64–68). This dynamic regulation modulates the balance between pro-inflammatory and regulatory immunity.

### 4.4 Immune cell infiltration across cancers

**Figure 1:**
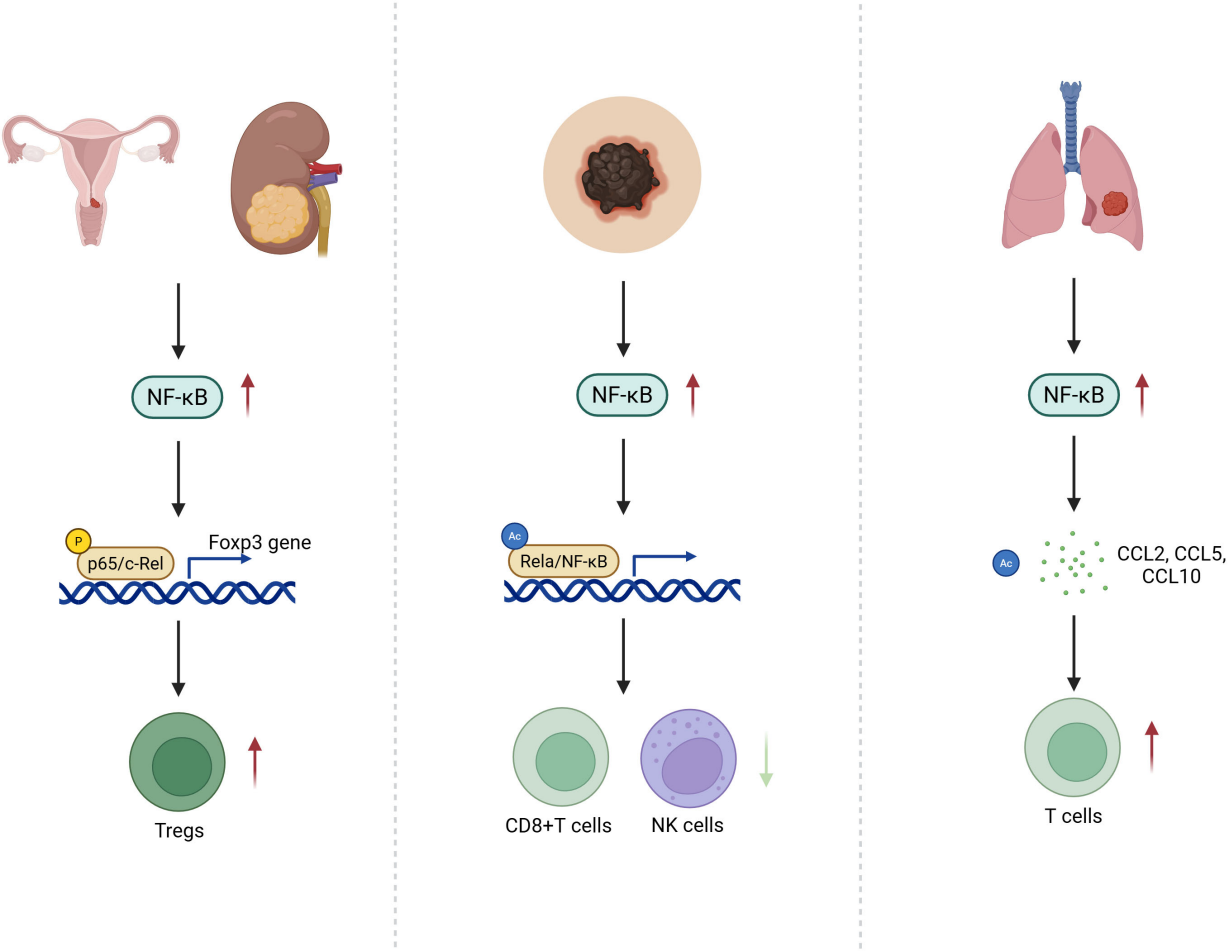
PTM-Encoded Divergence of NF-κB Signaling in T Cell Infiltration Across Cancer Types: Schematic illustration depicting the context-dependent effects of post-translationally modified NF-κB signaling on immune cell infiltration within the tumor microenvironment. In cervical and renal cancers, specific NF-κB PTM patterns enhance Treg recruitment, reinforcing an immunosuppressive milieu. In melanoma, aberrant PTM-driven NF-κB activity suppresses CD8^+^ T cell and NK cell infiltration, facilitating immune evasion. Conversely, in lung cancer, PTMs that favor pro-inflammatory NF-κB outputs lead to upregulation of chemokines such as CCL2, CCL5, and CXCL10, promoting effector T cell infiltration and antitumor immunity.

The influence of NF-κB signaling on immune cell infiltration is highly context dependent. In cervical and renal cancers, NF-κB promotes Treg accumulation and correlates with poor prognosis (61, 69). In melanoma, heightened NF-κB activity reduces CD8^+^ T and NK cell infiltration, exacerbating immune evasion (70). Conversely, in lung cancer, NF-κB activation upregulates chemokines such as CCL2, CCL5, and CXCL10, facilitating effector T cell infiltration and enhancing anti-tumor immunity (**Figure 1**) (23).

## 5 PTM-orchestrated NF-κB control of immune checkpoint networks

### 5.1 PD-1/PD-L1 Axis

The PD-1/PD-L1 axis represents a cornerstone of immune checkpoint regulation, where PD-L1 expression on tumor and immune cells suppresses T cell–mediated cytotoxicity. PD-1 functions to suppress autoimmunity. Cancer cells express PD-L1, which binds to PD-1 on the surface of T cells, thereby inhibiting T cell activation and leading to cancer immune evasion. This mechanism has been confirmed in various malignancies, including lung cancer, melanoma, glioma, and breast cancer (71). NF-κB acts as a master regulator of PD-L1, not only by direct promoter binding but also through cytokine-mediated amplification. Post-translational modifications (PTMs) of NF-κB subunits—including phosphorylation of p65 at Ser536 and acetylation at Lys310—enhance its transcriptional activity on the CD274 (PD-L1) promoter in diverse cancers such as NSCLC and gastric carcinoma (72–75).

In parallel, NF-κB indirectly upregulates PD-L1 by inducing pro-inflammatory cytokines including TNF-α, IL-6, and IFN-γ, which activate JAK/STAT pathways that synergize with NF-κB signaling. For instance, TNFR-mediated NF-κB activation in breast, ovarian, lung, and pancreatic tumors promotes robust PD-L1 expression (13, 76–78). IL-6–driven STAT3 activation can also potentiate NF-κB nuclear localization, reinforcing PD-L1 transcription in hepatocellular carcinoma and ovarian cancer.

**Figure 2:**
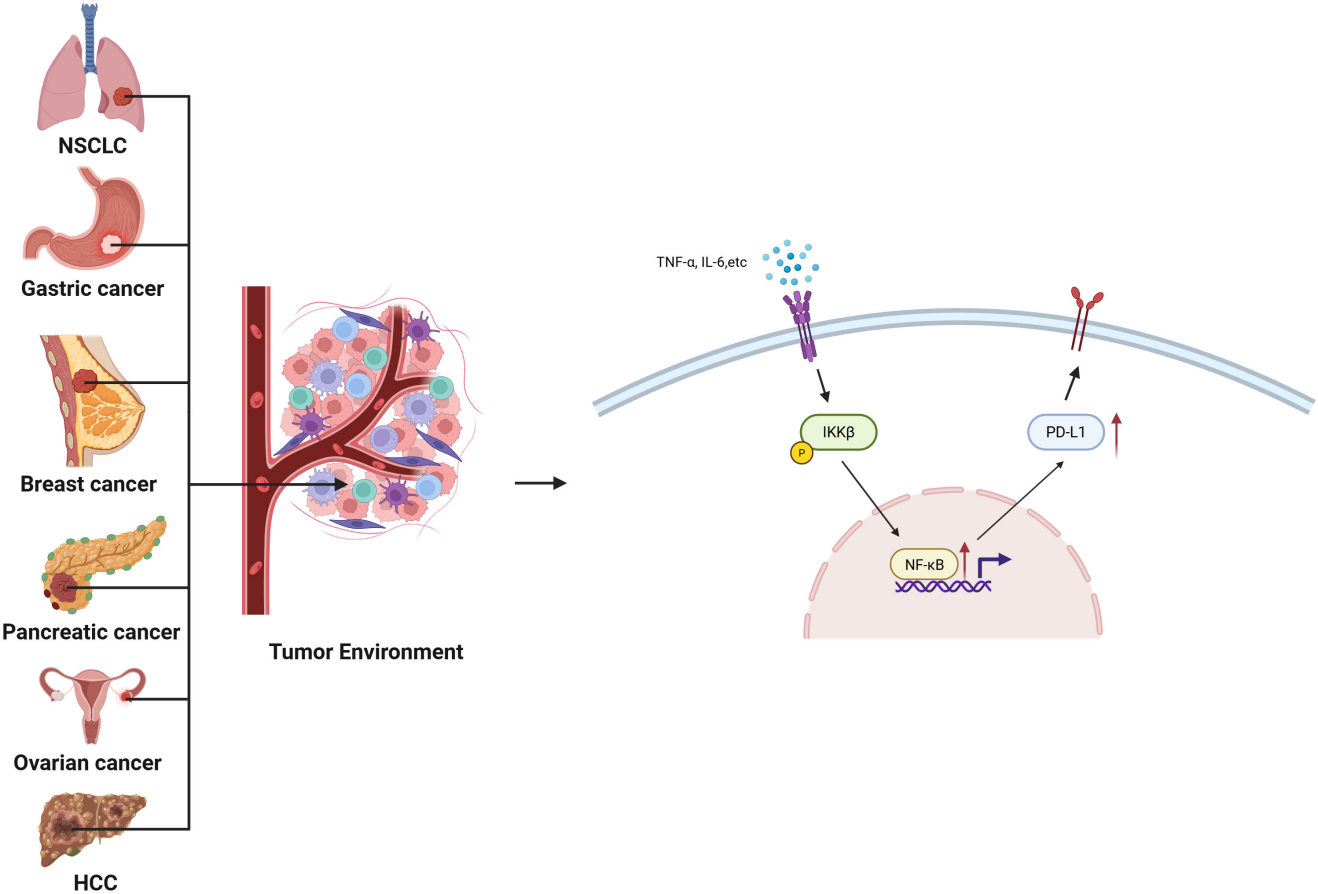
PTM-Tuned Mechanisms of NF-κB-Mediated PD-L1 Upregulation Across Cancers: Schematic illustration summarizing how PTM-activated NF-κB directly binds κB elements in the PD-L1 promoter and indirectly enhances PD-L1 expression via cytokine induction (e.g., TNF-α, IL-6, IFN-γ). These transcriptional and paracrine mechanisms converge to promote immune evasion and therapy resistance in NSCLC, gastric, breast, ovarian, pancreatic, and liver cancers.

PD-L1 expression not only suppresses CTL function by inhibiting IFN-γ and TNF-α production, but also contributes to T cell exhaustion and impaired proliferation. Moreover, NF-κB-mediated PD-L1 upregulation facilitates M2 macrophage polarization and Treg recruitment, reinforcing immune evasion (79). Notably, certain PTM patterns—such as SUMOylation of IκB kinase or hyperacetylation of RelA—have been linked to heightened PD-L1 transcriptional output. Thus, the PTM context of NF-κB activation critically shapes the magnitude and persistence of immune checkpoint induction (**Figure 2**).

### 5.2 CTLA-4

CTLA-4 is an inhibitory receptor expressed on activated T cells and constitutively on Tregs. While NF-κB does not directly bind the CTLA-4 promoter, it regulates CTLA-4 expression indirectly through the induction of Foxp3—the transcription factor essential for Treg lineage commitment. PTM-driven NF-κB signaling, particularly c-Rel phosphorylation and nuclear acetylation, enhances Foxp3 transcription, stabilizing the suppressive phenotype of Tregs and promoting CTLA-4 surface expression (80–84). CTLA-4 primarily exerts its immunosuppressive effects through Treg-mediated mechanisms, as reported in non-small cell lung cancer (NSCLC) and melanoma (85).

### 5.3 LAG-3

LAG-3 is expressed on exhausted T cells and Tregs, contributing to T cell inhibition upon engagement with MHC-II. Though direct transcriptional control by NF-κB remains poorly defined, PTM-enhanced NF-κB signaling induces cytokines such as IL-10, IL-27, and IL-12, which indirectly promote LAG-3 upregulation and functionality (86, 87). his regulatory axis reflects the broader role of NF-κB in coordinating the cytokine milieu that shapes immune checkpoint expression.

### 5.4 IDO1

IDO1 metabolizes tryptophan into kynurenine, leading to T cell anergy and immunosuppression. Its expression is transcriptionally controlled by the noncanonical NF-κB pathway via p52/RelB complexes. PTMs such as NIK ubiquitination and RelB methylation enhance nuclear translocation and IDO1 promoter binding (88–90). Additionally, IL-6/STAT3 signaling amplifies this effect, establishing a robust immunosuppressive feedback loop. IDO1-mediated tolerance further suppresses dendritic cell activation and impairs effector T cell responses (91). In esophageal cancer, IDO1 promotes tumor progression by facilitating the nuclear translocation of NF-κB and its binding to the CXCL10 promoter, thereby regulating the expression of the chemokine C-X-C motif ligand 10 (CXCL10) (92).

Together, these findings underscore the multilayered control exerted by NF-κB—both direct and cytokine-driven—over a spectrum of immune checkpoints. While PD-L1 and IDO1 are under direct transcriptional regulation by PTM-sensitive NF-κB complexes, CTLA-4 and LAG-3 are governed indirectly via modulation of Tregs and cytokine circuits (**Table 2**). Targeting the PTM machinery that tunes NF-κB activity may represent a novel strategy to destabilize immune checkpoints and enhance immunotherapy efficacy.

**Table 2:** Overview of immune checkpoint molecules regulated by PTM-governed NF-κB signaling in the tumor microenvironment.

Immune Checkpoint	Regulatory Mechanism by NF-κB	Key PTMs Involved	Immunological Impact	References
PD-L1/PD-1	Direct transcription by p65/c-Rel; cytokine-mediated enhancement via TNF-α, IL-6	p65 phosphorylation (Ser536); acetylation (Lys310); IKK SUMOylation	Promotes M2 polarization, Treg recruitment, and T cell exhaustion	(13, 74–78)
CTLA-4	Indirect regulation via Foxp3 transcription in Tregs	c-Rel phosphorylation and nuclear acetylation	Supports Treg lineage stability and immune suppression	(82, 84)
LAG-3	Indirect upregulation via NF-κB-induced cytokines (IL-10, IL-27)	NF-κB activation via cytokine feedback (IL-6, IL-1β)	Enhances Treg function and inhibitory T cell signaling	(87)
IDO1	Direct transcription via p52/RelB; amplified by IL-6–STAT3 pathway	RelB methylation; NIK ubiquitination	Inhibits T cell and DC function; reinforces tolerogenic TME	(90)

## 6 Targeting PTM-defined NF-κB circuits for cancer immunotherapy

### 6.1 Therapeutic targeting of NF-κB: inhibitors and mechanistic innovations

NF-κB remains an attractive but complex target in oncology, particularly in light of its dual role in immunity and tumor progression. Current therapeutic strategies include IKK inhibitors, direct NF-κB inhibitors, and next-generation proteolysis-targeting chimeras (PROTACs)—each shaped by distinct mechanisms of action and unique clinical challenges.

IKK inhibitors, such as BAY 11-7082, suppress canonical NF-κB activation by preventing IκBα phosphorylation (93). This results in reduced inflammatory cytokine production, dampened M2 polarization, and downregulation of immune checkpoints such as PD-L1. While promising in tumors with NF-κB-driven inflammation (e.g., colorectal, liver, HNSCC, and TNBC), these inhibitors are limited by systemic toxicity, off-target effects, rapid degradation, and compensatory activation of alternative pathways (e.g., PI3K, STAT3).

Direct NF-κB inhibitors, including Dehydroxymethylepoxyquinomicin (DHMEQ), covalently inhibit p65/p50 nuclear translocation (94, 95). This approach has demonstrated efficacy in reducing cytokine output and sensitizing tumors to immunotherapy across NSCLC, HCC, and melanoma models. Yet challenges with toxicity, resistance, and limited clinical data persist.

PROTACs represent a transformative approach by harnessing the ubiquitin-proteasome system to degrade target proteins. PROTAC molecules link a ligand for the protein of interest (e.g., NF-κB subunits, IKKβ, PD-L1) with a ligand for an E3 ubiquitin ligase (e.g., VHL, CRBN, MDM2) (96–99). This strategy offers prolonged target depletion and higher specificity compared to traditional inhibitors. PROTACs targeting NF-κB signaling have demonstrated the ability to degrade p65, IKKβ, and PD-L1, thereby reducing immunosuppressive cell populations such as MDSCs and Tregs and enhancing antitumor immunity. However, their large molecular size limits oral bioavailability, and clinical development remains at an early stage, with long-term safety yet to be fully evaluated. As summarized in **Table 3**, each class of NF-κB inhibitor presents unique therapeutic opportunities and challenges that must be balanced for optimal clinical translation.

**Table 3:** Overview of NF-κB inhibitors and their therapeutic applications in cancer.

Inhibitor	Target	Mechanism of Action	Cancer Types	Challenges
BAY 11-7082	IKKβ	Blocks IκBα phosphorylation, inhibits NF-κB nuclear translocation	HNSCC, colorectal cancer, liver cancer, TNBC	Immunosuppression, toxicity, rapid metabolism, resistance
DHMEQ	p65/p50	Covalent inhibition of NF-κB nuclear translocation	HCC, gastric cancer, NSCLC, melanoma, TNBC	Toxicity, resistance, limited clinical validation
PROTACs	NF-κB subunits, IKKβ, PD-L1	Ubiquitin-proteasome-mediated degradation	Pan-cancer	Low oral bioavailability, early-stage clinical development

#### 6.1.1 Challenges in clinical translation

Despite encouraging preclinical progress, the clinical translation of NF-κB-targeted therapies remains hampered by several intrinsic challenges—many of which are tightly intertwined with the pathway’s post-translational regulation. First, toxicity and immunosuppression pose major concerns. Systemic inhibition of NF-κB—particularly when indiscriminately targeting canonical components like IKKβ—can compromise host immunity and increase susceptibility to infection, given the pathway’s indispensable role in physiological immune responses. Fine-tuning NF-κB activity via modulation of specific PTMs (e.g., acetylation, SUMOylation) may offer a more selective approach to preserve immunological homeostasis. Second, target specificity remains elusive. NF-κB’s ubiquitous expression across normal and malignant tissues complicates efforts to develop tumor-selective inhibitors. A promising direction involves targeting tumor-specific PTM signatures of NF-κB subunits—such as context-specific acetylation or methylation patterns—that distinguish oncogenic NF-κB activity from its physiological roles. Third, therapeutic resistance frequently arises through compensatory signaling networks (e.g., PI3K-Akt, STAT3) or reprogramming of NF-κB PTM states that bypass inhibitor blockade. Rational combination strategies—such as co-targeting PTM-modifying enzymes (e.g., HDACs, methyltransferases) alongside immune checkpoints—may overcome adaptive resistance. Finally, delivery challenges—especially for large molecules like PROTACs or targeted epigenetic inhibitors—limit tissue penetration and bioavailability. Nanoparticle- or antibody-based delivery systems tailored to tumor-specific NF-κB PTM profiles may enhance pharmacokinetic properties while reducing off-target effects. Future therapeutic paradigms must therefore shift from global NF-κB inhibition toward precision rewiring of PTM-defined NF-κB circuits, enabling selective dismantling of tumor-promoting signals while preserving host immunity.

### 6.2 Leveraging PTM-regulated NF-κB for pan-cancer immunotherapy

#### 6.2.1 NF-κB Activity Scoring as a Predictive Biomarker

With the growing emphasis on personalized immunotherapy, transcriptome-based NF-κB activity scores have emerged as promising biomarkers to stratify patients and guide treatment selection. These scores, reflecting the cumulative activation of NF-κB-responsive genes, correlate with immunologically “hot” tumor microenvironments—marked by elevated IFN-γ signaling and high tumor mutational burden (TMB) (100).

In non-small cell lung cancer (NSCLC), high NF-κB activity has been associated with favorable responses to immune checkpoint inhibitors (ICIs) such as Atezolizumab (100). Notably, tumors with enriched NF-κB transcriptional signatures often exhibit elevated PD-L1 expression and T cell infiltration, indicating that NF-κB-high TMEs may be more amenable to immunotherapy. Incorporating this biomarker into clinical decision-making could enable rational combinations of ICIs with NF-κB or PTM-modulating agents, especially in tumors that exhibit inflamed yet immunosuppressive profiles.

#### 6.2.2 NF-κB as a therapeutic target to overcome immunotherapy resistance

Persistent NF-κB activation represents a critical barrier to durable immunotherapy responses, as it sustains immune checkpoint expression, promotes immune suppressive cell infiltration, and drives epithelial-mesenchymal transition (EMT). Importantly, several recent studies have demonstrated that targeting NF-κB—particularly its PTM-defined functional states—can reverse resistance and re-sensitize tumors to ICIs: BMS-345541, an IKKβ inhibitor, suppresses IFN-γ–induced PD-L1 expression, restoring T cell responsiveness (101). In prostate cancer, RelB-driven transcription enhances PD-L1/PD-1 signaling and mediates T cell dysfunction (102). In glioblastoma, the scaffolding protein PTRF/Cavin-1 promotes NF-κB–dependent PD-L1 upregulation, contributing to immune escape (103). In gastric cancer, NF-κB inhibition during EMT reduces PD-L1 expression and reverses immunotherapy resistance (11). In hepatocellular carcinoma, ependymin-related protein 1 (EPDR1) is an important tumor-intrinsic regulator of PD-L1 expression and tumor immune evasion. It promotes PD-L1 expression and immune escape by inhibiting TRIM21-dependent ubiquitination of NF-κB (15). In pancreatic ductal adenocarcinoma (PDAC), phosphorylation mediated by Polo-like kinase 1 (Plk1) suppresses NF-κB activity. Inhibition of Plk1 significantly increases NF-κB phosphorylation at Ser468, reactivating anti-tumor immunity and restoring sensitivity to immune checkpoint blockade, particularly anti–PD-L1 therapy (11). Additionally, in PDAC, targeting HDAC5-mediated deacetylation enhances NF-κB activity, thereby improving the efficacy of anti–PD-1 therapy (13) (**Table 4**). These findings underscore NF-κB as a convergent node in multiple resistance pathways. More importantly, targeting tumor-specific PTMs that enhance NF-κB’s immunosuppressive functions may unlock new therapeutic windows across refractory tumor types.

**Table 4:** Overview of the impact of targeting PTM-related NF-κB on cancer immunotherapy.

Tumours	Mechanism	Effect	Reference
HCC	Inhibiting ubiquitination of NF-κB	promotes PD-L1 expression and immune escape	(15)
PDAC	NF-κB phosphorylation	restoring sensitivity to anti–PD-L1 therapy	(11)
PDAC	deacetylation	improving anti–PD-1 therapy	(13)

## 7 Conclusions and future directions

NF-κB stands at the nexus of inflammation and immunity, orchestrating the transcriptional programs that shape tumor–immune interactions across malignancies. As a central regulator of both innate and adaptive responses, NF-κB controls cytokine networks, immune cell polarization, antigen presentation, and immune checkpoint expression—collectively sculpting the immunological tone of the TME. Yet, these functions are not uniform; rather, they are profoundly context-dependent and post-translationally encoded.

Emerging evidence reveals that the biological fate of NF-κB is largely dictated by PTMs—including phosphorylation, acetylation, methylation, ubiquitination, and SUMOylation—which modulate its activation threshold, subcellular localization, DNA-binding specificity, and crosstalk with co-factors. This PTM-defined plasticity explains the divergent immunological outcomes of NF-κB activation across different tumor types and microenvironmental contexts. Moving forward, mapping the PTM landscape of NF-κB in cancer will be essential to design selective and tumor-specific intervention strategies. From a translational perspective, PTM-informed NF-κB inhibition offers several therapeutic opportunities: 1) In inflammation-driven cancers such as colorectal, gastric, and pancreatic cancer, targeting PTM-mediated NF-κB hyperactivation may suppress immune evasion and prevent malignant transformation. 2) In checkpoint-resistant tumors (e.g., prostate, glioblastoma, TNBC), PTM-specific inhibitors or PROTAC-based degraders may re-sensitize tumors by downregulating PD-L1, IDO1, and immunosuppressive myeloid populations. 3) In immunologically “hot” tumors with high NF-κB activity, integrating NF-κB inhibition with ICIs may amplify cytotoxic responses while mitigating immune exhaustion. Technological advances will accelerate this field. AI-guided PTM target discovery, NF-κB activity scoring, and spatial transcriptomics will aid patient stratification and drug repurposing. Moreover, organoid models, intravital imaging, and CRISPR-engineered PTM-site mutants will facilitate functional dissection of NF-κB circuits within complex TMEs.

**Figure 3:**
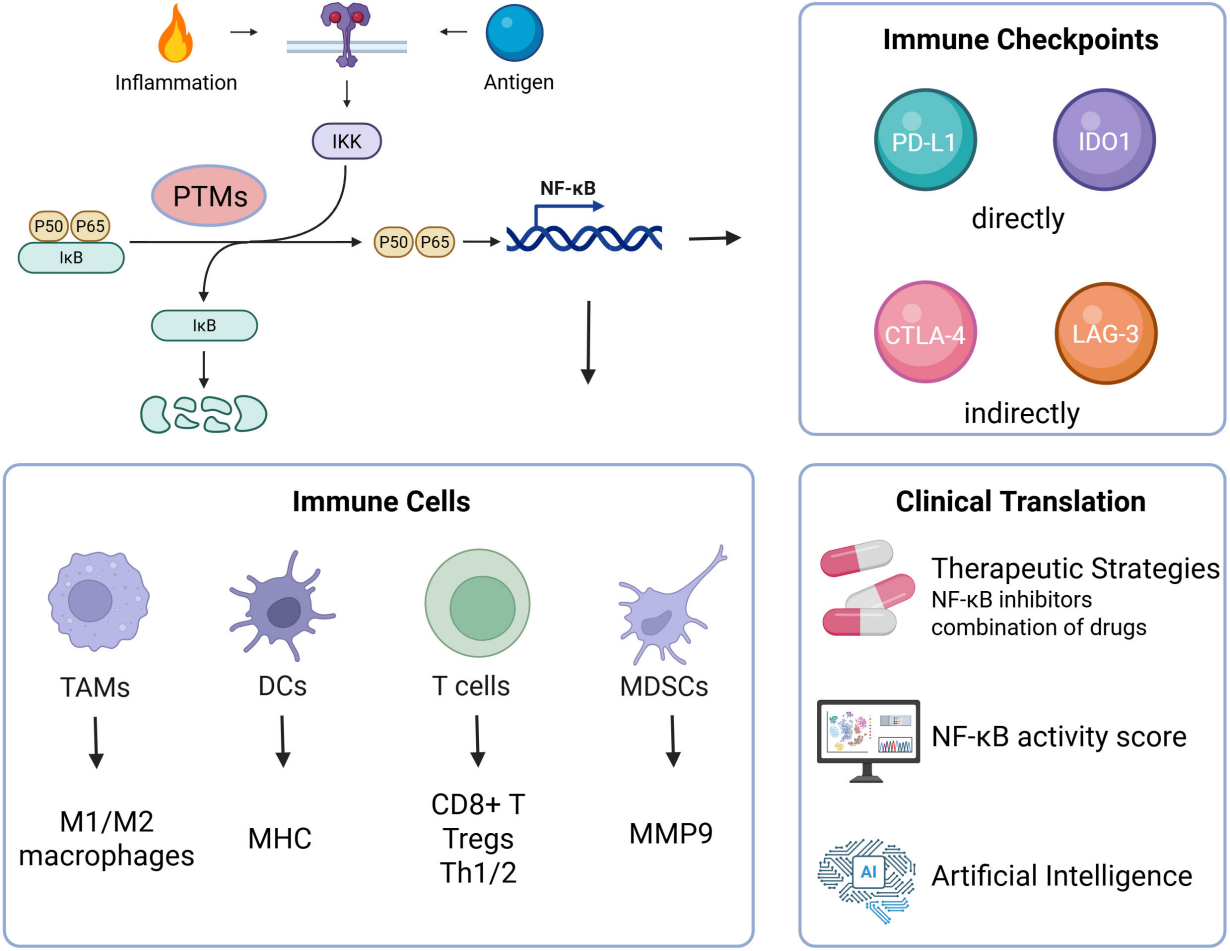
Schematic overview of NF-κB-mediated immune remodeling and translational therapeutic strategies: Upon activation by inflammatory or antigenic signals, NF-κB undergoes PTM-defined modulation to regulate key immune subsets—including TAMs, DCs, T cells, and MDSCs—and to orchestrate the expression of immune checkpoints. These effects converge to sustain an immunosuppressive microenvironment and promote tumor progression. Therapeutic strategies targeting NF-κB—ranging from selective inhibitors and PROTACs to combination regimens and computational tools such as activity scoring and AI-enabled modeling—offer promising avenues for precision immunotherapy.

Ultimately, the future of NF-κB-targeted therapy lies not in global suppression, but in the precision rewiring of PTM-governed NF-κB axes—disrupting its pathological immunosuppressive programs while preserving physiological immunity. As depicted in **Figure 3**, a refined understanding of these pathways holds the key to next-generation combination immunotherapies and the realization of personalized cancer care.
